# Phenotypic and transcriptomic characterization of canine myeloid-derived suppressor cells

**DOI:** 10.1038/s41598-019-40285-3

**Published:** 2019-03-05

**Authors:** Michelle R. Goulart, Sabina I. Hlavaty, Yu-Mei Chang, Gerry Polton, Anneliese Stell, James Perry, Ying Wu, Eshita Sharma, John Broxholme, Avery C. Lee, Balazs Szladovits, Mark Turmaine, John Gribben, Dong Xia, Oliver A. Garden

**Affiliations:** 1 Royal Veterinary College, London, UK; 2 School of Veterinary Medicine, University of Pennsylvania, Philadelphia, PA USA; 3 North Downs Specialist Referrals, Surrey, UK; 4 Wellcome Centre for Human Genetics, University of Oxford, Oxford, UK; 5 Perelman School of Medicine, University of Pennsylvania, Philadelphia, PA USA; 6 Division of Bioscience, University College London, London, UK; 7 Barts Cancer Institute, Queen Mary University of London, London, UK; 8 Present Address: Barts Cancer Institute, Queen Mary University of London, London, UK

## Abstract

Myeloid-derived suppressor cells (MDSCs) are key players in immune evasion, tumor progression and metastasis. MDSCs accumulate under various pathological states and fall into two functionally and phenotypically distinct subsets that have been identified in humans and mice: polymorphonuclear (PMN)-MDSCs and monocytic (M)-MDSCs. As dogs are an excellent model for human tumor development and progression, we set out to identify PMN-MDSCs and M-MDSCs in clinical canine oncology patients. Canine hypodense MHC class II^−^CD5^−^CD21^−^CD11b^+^ cells can be subdivided into polymorphonuclear (CADO48A^+^CD14^−^) and monocytic (CADO48A^−^CD14^+^) MDSC subsets. The transcriptomic signatures of PMN-MDSCs and M-MDSCs are distinct, and moreover reveal a statistically significant similarity between canine and previously published human PMN-MDSC gene expression patterns. As in humans, peripheral blood frequencies of canine PMN-MDSCs and M-MDSCs are significantly higher in dogs with cancer compared to healthy control dogs (PMN-MDSCs: p < 0.001; M-MDSCs: p < 0.01). By leveraging the power of evolution, we also identified additional conserved genes in PMN-MDSCs of multiple species that may play a role in MDSC function. Our findings therefore validate the dog as a model for studying MDSCs in the context of cancer.

## Introduction

Myeloid-derived suppressor cells (MDSCs) comprise a functionally distinct phenotype of innate immune cells that play an important role in the immune dysregulation characteristic of cancer^1–4^. Recent years have witnessed an increasing recognition of the clinical relevance of MDSCs. Accumulation of these cells has been reported in practically all human cancers, and increased frequencies of circulating MDSCs have been correlated with poor prognosis, offering a biomarker of clinical outcome in a variety of cancer histotypes^5^.

Generated by pathological subversion of polymorphonuclear (PMN) and monocytic (M) differentiation and activation pathways in the context of chronic inflammatory conditions and cancer, MDSCs represent a heterogeneous population of two major subsets, PMN-MDSCs and M-MDSCs, which are identified by a combination of multiple lineage markers. In mice, PMN-MDSCs are defined as CD11b^+^Ly6G^+^Ly6C^lo^ cells, while M-MDSCs are defined as CD11b^+^Ly6G^−^Ly6C^+^ cells. In humans, PMN-MDSCs are characterized as CD11b^+^CD14^−^CD15^+^ or CD11b^+^CD14^−^CD66b^+^ cells, while M-MDSCs are CD11b^+^CD14^+^HLA-DR^−/lo^ CD15^−^ cells. A third group comprising immature myeloid progenitors has also been described as Lin^−^(CD3/14/15/19/56)/^−^HLA-DR^−^/CD33^+^ cells^4,6–8^.

Although a number of pivotal mechanistic studies on the pathobiology of cancer have been performed using the mouse as a model for humans, there is an unmet need for animal models that better recapitulate human cancer to investigate novel therapeutic targets, including cellular targets such as MDSCs^9,10^. Canine malignancies have already been recognized as strong comparative models for several human cancers^11,12^. Dogs spontaneously develop a variety of tumors that share many features with human cancer, including clinical, pathological, and molecular characteristics^11–13^. Furthermore, dogs have an intact immune system that allows faithful recapitulation of the tumor microenvironment and circulating regulatory T cells of human patients^11,14^. Of note, a number of drugs utilized in veterinary medicine were originally developed for human use, further emphasizing the bilateral benefits of the One Health approach to both dogs and humans alike^11–13^. The contributions of the comparative oncology field thus far therefore raise the question of whether dogs with spontaneous tumors may also shed insight into MDSC biology.

To date, two seminal studies described MDSCs in a variety of cancer histotypes in dogs^15,16^, but many questions remain unanswered. Although these studies identified the existence of this suppressive myeloid cell population in the peripheral blood of dogs, this early work did not characterize the two subsets of MDSCs, an essential prerequisite to their investigation in canine models of human cancer. The current study therefore set out to characterize MDSC subsets in tumor-bearing dogs, hypothesizing that their cellular phenotype and transcriptomic signatures would reflect those of both human and murine MDSC subsets. We identified two distinct myeloid cell populations in the circulating blood of tumor-bearing dogs with similar phenotypic, functional, and transcriptomic characteristics to human and murine PMN-MDSCs and M-MDSCs. Capitalizing on the power of a comparative evolutionary approach, we characterized the cellular and transcriptomic phenotype of both PMN-MDSCs and M-MDSCs. We identified five transcripts that are expressed at high levels by canine, human, and murine PMN-MDSCs, yielding novel insights into fundamentally conserved PMN-MDSC gene expression patterns spanning multiple evolutionary taxa.

## Materials and Methods

### Study population and sample collection

Peripheral blood samples were collected from dogs with cancer or non-neoplastic inflammatory diseases recruited at the Royal Veterinary College (RVC), North Downs Specialist Referrals (NDSR), and Fitzpatrick Referrals (Oncology and Soft Tissue; FR) in the United Kingdom, and the School of Veterinary Medicine at the University of Pennsylvania (Penn Vet) in the United States of America. Healthy control dogs were also recruited at the RVC and Penn Vet, defined by an absence of clinically significant findings following a detailed history and physical examination performed by a veterinarian or veterinary nurse. Inflammatory control dogs included those with infectious or immune-mediated disease, in which neoplasia was ruled out by relevant diagnostic tests, including imaging of the thorax and/or abdomen. Forty-one tumor-bearing, 37 inflammatory, and 31 healthy dogs were recruited at the RVC; 51 tumor-bearing dogs were recruited at NDSR; five tumor-bearing dogs were recruited at FR; and 21 tumor-bearing, three inflammatory, and 25 healthy dogs were recruited at Penn Vet.

Tumor burden was classified as follows: For B cell lymphoma, patients were grouped based on the World Health Organization staging system, with stage I/II defined as low burden and stage III-IV defined as high burden. In patients with solid non-lymphoid tumors, a low burden tumor was defined as one whose sum of the longest diameters of the lesions was smaller than 5 cm without evidence of lymph nodal and/or distant metastasis. A high burden tumor was defined as one whose sum of the longest diameters of lesions was greater than 5 cm, with or without evidence of lymph nodal and distant metastasis. This study was approved by the RVC’s Clinical Research Ethical Review Board (URN 2014 1285), the University of Pennsylvania’s Institutional Animal Care and Use Committee, and Penn Vet’s Privately Owned Animal Protocol Committee; all samples were collected and processed in accordance with the relevant guidelines and regulations. A peripheral blood sample was collected aseptically from each dog from the jugular or lateral saphenous vein into one or more EDTA tubes after informed consent was granted by the owner of each dog. All blood was processed within 48 hours of collection.

### Preparation of PBMCs and PMN cell isolation

MDSCs were isolated from the mononuclear fraction following density gradient centrifugation as follows. Blood was diluted with an equal volume of sterile Dulbecco’s Phosphate-Buffered Saline (DPBS; Corning Cellgro, Tewksbury, MA, USA) containing 2% v/v fetal bovine serum (FBS; Hyclone, Logan, UT, USA), and gently layered over Histopaque-1077^®^ (Sigma-Aldrich, St. Louis, MO, USA). Samples were centrifuged at 400 *g* for 30 minutes, with acceleration and deceleration set to zero. The peripheral blood mononuclear cell (PBMC) layer was washed twice with Roswell Park Memorial Institute (RPMI)-1640 medium (Life Technologies, Carlsbad, CA, USA) containing 10 mM HEPES, 2 mM L-glutamine (Life Technologies), 100 units/mL penicillin-streptomycin (Life Technologies), 10% FBS, and 50 μM 2-mercaptoethanol (Life Technologies) (complete medium), before re-suspension in DPBS containing 10% v/v FBS and staining for analytical flow cytometry or flow-assisted cell sorting (FACS™). PMNs were isolated from the remaining cell fraction after removal of the mononuclear cells and treatment with Red Blood Cell (RBC) Lysis Buffer (Multi-Species; Thermo Fisher Scientific, San Diego, CA, USA) according to the manufacturer’s instructions. After RBC lysis, cells were washed and re-suspended in DPBS-10% v/v FBS for staining.

### Flow cytometry and cell sorting

For MDSC analysis, PBMCs were labelled with a cocktail of monoclonal antibodies (mAb) for 30 minutes at 4 °C according to the respective manufacturer’s protocol. The cocktail comprised APC-conjugated anti-dog MHC II (clone YKIX334.2; Thermo Fisher Scientific), PE-conjugated anti-dog-CD5 (clone YKIX322.3; Bio-Rad, Hercules, CA, USA), PE-conjugated anti-dog CD21 (clone CA2.1D6; Bio-Rad), PE-Alexa647-conjugated anti-human CD14 (clone TUK4; Bio-Rad), PE-Cy7-conjugated anti-dog PMN leukocyte antigen (antigen unknown, clone CADO48A; University of Washington, Pullman, WA, USA), and Alexa Fluor 488-conjugated anti-mouse CD11b (clone M1/70; Thermo Fisher Scientific). PMNs from tumor-bearing or healthy dogs were identified as CADO48A^+^ cells isolated from the RBC-containing pellet. Antibody-labeled cells were washed twice and re-suspended in DPBS-10% v/v FBS, then incubated with 4′,6-diamidino-2-phenylindole (DAPI; BioLegend, San Diego, CA, USA) at room temperature for 10 minutes prior to analysis.

Flow cytometric data were acquired using a FACSCanto II flow cytometer (Becton-Dickinson (BD); Franklin Lakes, NJ, USA) and analyzed using FlowJo^®^ software, version 10.3 (Tree Star, Ashland, OR, USA). Cell sorting was performed using a BD FACSAria III or a BD FACSAria Fusion in the case of dogs recruited at the RVC, and a BD FACSAria II in the case of dogs recruited at Penn Vet. Monocytes were isolated from the PBMC layer and identified as CD5^−^CD21^−^MHCII^+^CD11b^+^CD14^+^ cells (H MONOs, healthy donor; C MONOs, tumor-bearing donor), while PMNs were isolated from the hyperdense pellet and identified as CD5^−^CD21^−^MHCII^−^CD11b^+^CADO48A^+^ cells (H PMNs, healthy donor; C PMNs, tumor-bearing donor). MDSCs were isolated from the PBMC layer, with M-MDSCs identified as CD5^−^CD21^−^MHCII^−^CD11b^+^CD14^+^CADO48A^−^ cells, and PMN-MDSCs identified as CD5^−^CD21^−^MHCII^−^CD11b^+^ CD14^−^CADO48A^+^ cells.

### Analysis of MDSC frequencies

Frequencies of MDSCs isolated from peripheral blood were reported as median [25^th^, 75^th^ percentiles]. A linear mixed effects model was used to assess differences between PMN-MDSCs and M-MDSCs, and among tumor-bearing, inflammatory disease and healthy dogs, and specific contrasts were constructed to compare high and low burden tumors. Frequencies of MDSCs were log transformed, ln (0.1+ frequency), prior to the analysis.

### Cytocentrifuge spin preparation

A Shandon Cytospin 2 centrifuge (Sandon Southern Products Ltd., Astmoor, UK) was used to deposit cells onto Shandon cytoslides (Thermo Sandon Limited, UK). The slides were dried, stained with Modified Wright’s (Siemens Hematek Stain Pak, NY, USA) using a Hematek automated stainer (Siemens, Tarrytown, NY, USA) and examined with a BX50 microscope (Olympus, Tokyo, Japan). Images were captured with a SC50 camera and edited with CellSens^®^ software (Olympus; Southend-on-Sea, UK).

### Electron microscopy and ER dilation scoring

PMN-MDSCs and PMNs were isolated from two tumor-bearing dogs, while monocytes were isolated from one tumor-bearing dog. Cells were fixed in 2% w/v paraformaldehyde and 1.5% w/v glutaraldehyde in 0.1 M sodium cacodylate for 24 hours at 3 °C. After washing in 0.1 M sodium cacodylate twice, each time for 30 minutes, cells were embedded in 2% w/v agarose. Specimens were then dehydrated in a graded ethanol-water series, before being immersed in agar resin. The specimens were left in fresh agar resin for eight hours, before being hardened for 48 hours at 60 °C. Representative areas were selected and ultra-thin sections cut at 70–80 nm using a diamond knife in an Ultracut S microtome (Reichert Technologies; Munich, Germany). Sections were collected on 200 mesh copper, stained with lead citrate, and viewed with a 1010 transition electron microscope (Jeol; Peabody, MA, USA). Images were recorded using an Orius CCD camera (Gatan; Pleasanton, CA, USA). Our approach was adopted from a published protocol^17^, reviewing at least 100 cells per sample. The proportion of visible cytoplasmic area per cell containing dilated ER was estimated, scores 1, 2, and 3 representing areas of up to one third, more than one third but less than two thirds, and more than two thirds respectively.

### T cell suppression assay

Proliferation assays were performed in duplicate, when cell numbers allowed, in 96-well, flat bottom culture plates pre-coated with anti-dog CD3 mAb (clone CA17.2A12; Bio-Rad), applied as a 5 µg/ml solution for two hours at 37 °C before washing. An aliquot of 100,000 healthy PBMCs was deposited into each well, alone or with either PMNs from tumor-bearing or healthy dogs, or PMN-MDSCs, at a 1:1 ratio. Anti-CD28 mAb (clone 1C6; ThermoFisher Scientific) was added to each well at a final concentration of 2.5 µg/ml. Plates were incubated at 37 °C for 72 hours in an atmosphere of 5% carbon dioxide, before staining of the cells for analytical flow cytometry on a BD FACSCanto II. Responder PBMCs and co-cultured cells, if applicable, were harvested and stained with eBioscience fixable viability dye (eFluor780, Thermo Fisher Scientific) for 20 minutes at room temperature, followed by a 30 minute incubation at 4 °C with a cocktail comprising of anti-dog CD5 (as above) and AlexaFluor647-conjugated anti-dog CD8 mAb (YCATE55.9; Bio-Rad). The cells were then fixed and permeabilized (Foxp3/Transcription Factor Staining Buffer Set; Thermo Fisher Scientific) prior to staining with pacific blue-conjugated anti-mouse/rat Ki-67 (SolA15; Thermo Fisher Scientific) for 30 minutes at 4 °C. A linear mixed effects model was used to analyze suppression assay results. Normality of the residuals were inspected using histograms and Shapiro-Wilk normality test. The analyses were carried out in R using lme4 (Version 1.1.17), lmerTest (Version 3.0.1) and multcomp (Version 1.4.8) packages.

### RNA extraction

FACS™ was used to isolate MDSC subsets from dogs with cancer, alongside PMNs and monocytes from both tumor-bearing and healthy dogs. Cells were centrifuged at 600 *g* for five minutes at 4 °C, re-suspended in RNA-Bee (Amsbio; Abingdon, UK), and stored at −80 °C until extraction. RNA extraction was performed using the Direct-zol RNA MicroPrep Kit (Zymo Research; Irvine, CA, USA) according to the manufacturer’s protocol. RNA was stored at −80 °C.

### Library preparation and sequencing

RNA sequencing (RNA-Seq) libraries were prepared and sequenced by the Oxford Genomics Centre at the Wellcome Centre for Human Genetics, using the protocol for low-input RNA library preparation with Smart-seq2^18^. Briefly, full-length cDNA was generated from 0.6–1.0 ng of total RNA. Illumina libraries were prepared from cDNA samples using the Nextera XT DNA Library Prep Kit (Illumina, San Diego, CA, USA) and PCR-amplified with in-house indexing primers^19^. All samples were sequenced on the Illumina HiSeq4000 platform, with 75 bp paired-end sequencing.

### Read processing and expression quantification

Reads were trimmed for Nextera, Smart-seq2 and Illumina adapter sequences using skewer-v0.1.125^20^. Trimmed read pairs were aligned to a modified reference genome comprising the canine genome *Canis familiaris* CanFam3.1 and External RNA Controls Consortium (ERCC) spike-in mix sequences (ThermoFisher), using HISAT2 version 2.0.4^21^ with default parameters. Duplicate reads were marked using MarkDuplicates.jar implemented in Picard tools v1.92 (http://broadinstitute.github.io/picard/, February 2018). Binary Alignment Map (BAM) alignments were name-sorted with Samtools version 1.1^22^. Reads mapping uniquely to exons of genes annotated in Ensembl release 81^23^ were counted using featureCounts^24^ implemented in subread-v1.5^25^.

### Data quality control and differential expression analysis

Alignment metrics were calculated using CollectRnaSeqMetrics from Picard tools for both full BAM files, and BAM files with potential PCR duplicates were marked. Additional metrics were calculated and downstream analysis performed with custom R scripts using R core tools^26^, v3.1.0 and v3.4.2 respectively. Differentially expressed genes were identified for each comparison using edgeR v3.20.4^27,28^. Genes with significant differential expression and a false discovery rate (FDR) ≤0.05 in each comparison were used for gene-ontology enrichment analysis using GOseq v1.22.0^29^.

Gene enrichment comparisons were plotted on Venny 2.1.0 (http://bioinfogp.cnb.csic.es/tools/venny/). Ingenuity Pathway Analysis (IPA) (Qiagen, Hilden, DE) was used for interpretation of the RNA-Seq data, using differentially expressed genes whose fold change (FC) ≥2 and FDR ≤ 0.05. In pathway enrichment analysis, p-values were calculated by right-tailed Fisher’s exact tests, which indicate the probability of association of molecules from the dataset with the canonical pathway by random chance alone. The Z-score was used to quantitatively compare the dataset with the canonical pathway patterns, taking into account the activation state of one or more key molecules when the pathway was activated, as well as the molecules’ causal relationships with each other. Pathways with p ≤ 0.05 in the enrichment analysis were considered enriched, while the activation states of pathways were determined when |Z-score| ≥ 2. Heatmaps illustrating the RNA-Seq dataset were created in Morpheus (https://software.broadinstitute.org/morpheus/, Broad Institute), using only those genes expressed in every patient and cell type with transcripts per million (TPM) values of over 10. Clustering of the rows and columns in the heatmap comparing transcriptomic signatures of different cell types was performed using the 1-Spearman rank correlation metric as the measure of dissimilarity. Box-and-whisker plots were drawn and all transcriptomic analyses were undertaken in R v3.4.2 (The R Foundation, Vienna, AT), using R packages lattice v0.20.35 and ggplot2 v2.2.1. R was also used to create principal component analysis (PCA) and volcano plots.

### Reverse transcriptase-quantitative PCR

RNA was converted into cDNA using the High-Capacity RNA-to-cDNA Kit (ThermoFisher Scientific), following the manufacturer’s protocol. RT-qPCR reactions were prepared according to the manufacturer’s protocol, using TaqMan Fast Advanced Master Mix (ThermoFisher Scientific). Primers, all purchased from ThermoFisher Scientific, recognized hypoxanthine phosphoribosyltransferase 1 (HPRT1; Cf02690456_g1), ribosomal protein L13a (RPL13A; Cf04947268_gH), lactoferrin (LTF; Cf02649397_m1), lipocalin 2 (LCN2; Cf02667820_m1), cathelicidin (CAMP; Cf02626391_m1), erythrocyte band protein EPB41L3 (Cf02682517_m1), and the matrix metalloprotease MMP8 (Cf03649138_u1). The reactions were run on a Quant Studio 6 Flex Real-Time PCR System (ThermoFisher Scientific), using recommended cycle conditions for TaqMan Fast Advanced Master Mix: hold at 50 °C for 2 minutes, hold at 95 °C for 20 seconds, and 40 cycles of denaturing for 1 second at 95 °C, followed by annealing and extending for 20 seconds at 60 °C.

No-template and no-reverse transcriptase reactions were used as controls, verifying the specificity of the assay. PCR efficiency was determined by testing the primers across a 4 or 5-fold logarithmic dilution of cDNA template. The plot of Cq versus template concentration was used to calculate the slope, and amplification efficiency (E) of individual primers was determined by the equation ${\rm{E}}={10}^{-1/{\rm{slope}}}$. The most stable reference genes, HPRT1 and RPL13A, were selected from a set of tested candidate reference genes as determined by the qbase + (Biogazelle) implementation of geNorm. Relative expression of genes was determined by Pfaffl’s model,

$${\rm{Relative}}\,{\rm{Expression}}=\frac{{({{\rm{E}}}_{{\rm{target}}})}^{{{\rm{\Delta }}\mathrm{Cq}}_{{\rm{target}}}(control-sample)}}{{({{\rm{E}}}_{{\rm{ref}}})}^{{{\rm{\Delta }}\mathrm{Cq}}_{{\rm{ref}}}(control-sample)}}$$

where E_target_ is the efficiency of the target gene primer, E_ref_ is the efficiency of the reference gene primer, ΔCq_target_ is the difference in Cq of C PMNs and PMN-MDSCs for the target gene transcript, and ΔCq_ref_ is the difference in Cq of C PMNs and PMN-MDSCs for the reference gene transcript.

### Cross-species comparisons

Data used for the cross-species analysis of PMN-MDSCs were obtained from the authors^30^ and NCBI (GEO accession number GSE43254)^31^. Differential gene expression for murine data was evaluated in GEO2R (NCBI), using the Benjamin-Hochberg multiple comparisons correction and auto-detecting log transformation. Gene lists used to create Venn diagrams excluded genes with absolute FC < 2 and FDR > 0.05. Only those genes expressed in all three species were included in similarity score calculation. Gene lists for each species were ranked in the following order: FDR ≤ 0.05 and FC ≥ 2, FDR ≤ 0.05 and FC ≤ −2, FDR ≥ 0.05 and FC ≥ 2 and FDR ≥ 0.05 and FC ≤ −2, so that most significantly upregulated genes were at the top and the most significantly downregulated genes were at the bottom of the list, followed by non-statistically significant genes. Each cell-type-specific signature from dog was compared with respective human and murine signatures using the R package OrderedList v1.48.0^32^. This tool determines the number of shared elements in the first n elements of two lists and calculates a final similarity score, genes receiving more weight the closer they are to the top or bottom of the list. Similarity scores for n = 500 are reported. To assess the statistical significance of the similarity scores, the observed values were compared with a null distribution obtained by reshuffling the genes.

Pathway enrichment analysis was performed in IPA for the 44 commonly upregulated genes in human and canine PMN-MDSCs. Those pathways containing two or more genes (from the list of 44 shared genes) showing the strongest statistical significance were reported.

## Results

### Hypodense myeloid cell subsets accumulate in the peripheral blood of tumor-bearing dogs

**Figure 1:**
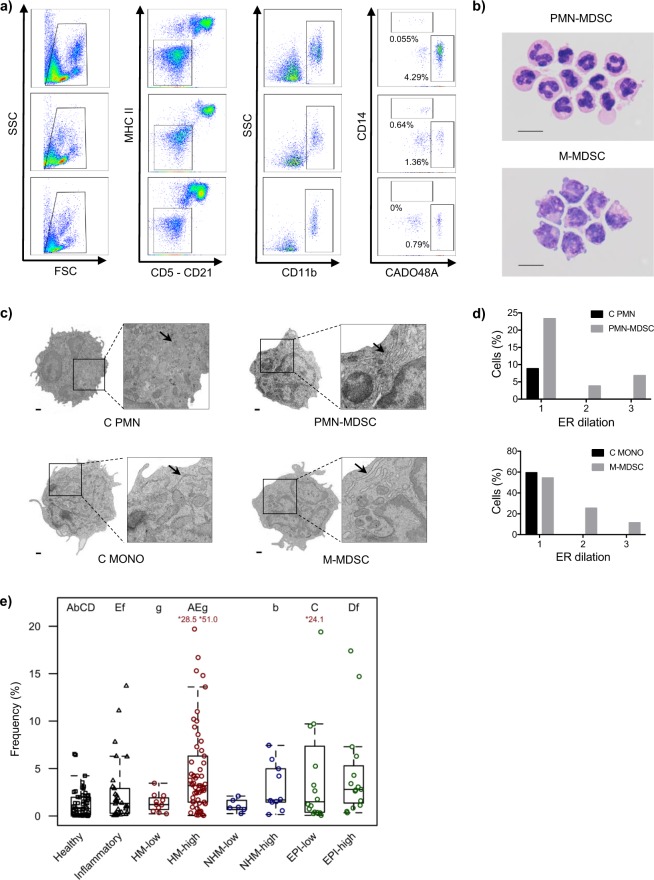
Two phenotypically distinct subsets of hypodense myeloid cells can be isolated from canine peripheral blood. (**a**) Gating strategy used to identify putative PMN-MDSC (CADO48A^+^CD14^−^) and M-MDSC (CADO48A^−^CD14^+^) subsets in the peripheral blood of dogs. Shown are representative examples from dogs that have PMN-MDSC frequencies (relative to total PBMCs) similar to the median values of all dogs in that group (cancer, top, median: 2.58%; inflammatory, middle, median: 1.33%; healthy, bottom, median: 0.78%). (**b**) Cytocentrifuge preparations of flow-sorted MDSCs with PMN (top) and MONO (bottom) morphology. Scale bars: 10 μm. (Images collected by B.S.). (**c**) Images obtained through electron microscopy analysis of cell morphology, with arrows denoting prominent ER. Scale bars: 500 nm, magnification 9700x, featured square magnification 3600x. (Images collected by M.T.). (**d**) C PMNs, PMN-MDSCs, C MONOs, and M-MDSCs were scored for the presence of dilated ER; the bar chart depicts the proportion of cells with each ER dilation score. (**e**) Box-and-whisker plots depicting frequency of PMN-MDSCs in peripheral blood of healthy and inflammatory controls as well as tumor-bearing dogs, grouped by cancer subtype (HM: hematopoietic mesenchymal/mesodermal, NHM: non-hematopoietic mesenchymal/mesodermal, EPI: epithelial) and burden (low vs high). Frequencies are expressed as a percentage of total PBMCs. A capital letter denotes that the two groups are significantly different with p < 0.001; a lowercase letter denotes a significant difference with p < 0.01. In the case of all box-and-whisker plots, the box shows the respective 25th and 75th percentiles, the line indicates the median value, and the whiskers stretch from the lowest to the highest data points still within 1.5 times the interquartile range of the respective lower and upper quartiles. Red values indicate patient frequencies that are off-scale. Each dot represents a single dog.

To test the hypothesis that phenotypically distinct hypodense myeloid cell subsets resembling PMN-MDSCs and M-MDSCs exist in canine blood, PBMCs were stained with a mAb panel reflective of the phenotypic markers used to identify human MDSC subsets^4,6,33^. Using a cascaded gating strategy as shown in Fig. 1a, hypodense myeloid cells resembling MDSCs, i.e. CD5^−^CD21^−^MHCII^−^CD11b^+^ cells, were stratified into two distinct subsets – one expressing CADO48A^+^, a canine specific neutrophil marker (putative PMN-MDSCs), and the other expressing CD14^+^ (putative M-MDSCs). Morphologic examination of cytocentrifuged preparations of these two myeloid populations supported their putative identity as MDSC subsets: the CADO48A^+^ cells resembled PMNs with segmented nuclei, while the CD14^+^ cells resembled monocytes with a large nucleus and vacuolar cytoplasm, reflecting MDSC images previously published in the human and murine literature^34–37^ (Fig. 1b). Parental gating analyses were also consistent with our view that these cells were MDSCs (Supplementary Fig. S1A).

We next interrogated the ultrastructure of these myeloid subsets, in particular to ask whether they exhibited features of ER stress, manifested through dilation, as has been described in human MDSC subsets^17^. Putative PMN-MDSCs, putative M-MDSCs, PMNs and MONOs were isolated from tumor-bearing dogs and scored for the presence of dilated ER. Although a small proportion of cancer PMNs and MONOs had mild ER dilation (see arrows), with a score of 1, only the putative PMN-MDSCs and putative M-MDSCs exhibited moderate to marked ER dilation (see arrows), with scores of 2 or 3 (Fig. 1c,d), further supporting their identity as MDSCs.

Since increased frequencies of MDSC subsets have been described in human cancer patients and tumor-bearing mice compared to individuals with non-neoplastic inflammatory diseases and healthy controls, we set out to determine whether this pattern could be observed in dogs. First, a global comparison of CADO48A^+^ and CD14^+^ CD5^−^CD21^−^MHCII^−^CD11b^+^ myeloid cell frequencies (relative to total PBMCs) in the peripheral blood of tumor-bearing *versus* control dogs revealed an increased frequency of the CADO48A^+^ myeloid population (putative PMN-MDSCs; 2.53% [0.93, 4.94]) in comparison to healthy dogs (0.78% [0.13, 1.95]; p = 1.8 × 10^−12^) and those with non-neoplastic inflammatory diseases (1.33% [0.31, 2.75]; p = 0.0024) (Supplementary Fig. S1B). The CD14^+^ myeloid population (putative M-MDSCs) was also increased in tumor-bearing dogs (0.20% [0.06, 0.57]) when compared to healthy control dogs (0.14% [0.05, 0.24]; p = 0.018) but not those with inflammatory disease (0.28% [0.12, 0.64]; p = 0.72) (Supplementary Fig. S1C).

Tumor-bearing dogs were then grouped according to broad tumor histotype (hematopoietic mesenchymal/mesodermal, HM; non-hematopoietic mesenchymal/mesodermal, NHM; and epithelial, EPI) and based on tumor burden (low *versus* high burden) (Fig. 1e). In the case of the putative PMN-MDSCs, dogs with high burden HM tumors had higher median frequency than those with low burden HM tumors (3.20% [1.46, 6.32] *versus* 1.20% [0.69, 1.91]; p = 0.0086); statistically significant differences in frequency with burden were not present for NHM tumors (1.69% [1.48, 4.79] *versus* 0.89% [0.72, 1.47]; p = 0.17) or EPI tumors (2.81% [1.37, 5.30] *versus* 1.50% [0.40, 6.31]; p = 0.28). Furthermore, dogs with a high tumor burden, regardless of histotype, had higher frequencies of these cells than healthy control dogs (HM: 3.20% [1.46, 6.32] *versus* 0.78% [0.13, 1.95], p = 1.5 × 10^−13^; NMH: 1.69% [1.48, 4.79] *versus* 0.78% [0.13, 1.95], p = 0.0034; EPI: 2.81% [1.37, 5.30] *versus* 0.78% [0.13, 1.95], p = 5 × 10^−6^). Dogs with high burden HM or EPI tumors also had higher frequencies of the putative PMN-MDSCs than those of non-neoplastic inflammatory control dogs (HM: 3.20% [1.46, 6.32] *versus* 1.33% [0.31, 2.75], p = 0.00015; EPI: 2.81% [1.37, 5.30] *versus* 1.33% [0.31, 2.75], p = 0.0091, respectively). Of dogs with low burden tumors, only those bearing EPI tumors had higher frequencies of these cells than healthy control dogs (1.50% [0.40, 6.31] *versus* 0.78% [0.13, 1.95], p = 0.00098), but not inflammatory control dogs (1.50% [0.40, 6.31] *versus* 1.33% [0.31, 2.75], p = 0.16). Similar differences were not apparent for putative M-MDSCs (Supplementary Fig. S1D). However, dogs with high burden as well as low burden EPI tumors had higher frequencies of putative M-MDSCs than healthy control dogs (high burden: 0.31% [0.09, 0.87] *versus* 0.14% [0.05, 0.24], p = 0.037; low burden: 0.21% [0.15, 0.86] *versus* 0.14% [0.05, 0.24], p = 0.01).

*In toto*, these findings added further credibility to our hypothesis that the myeloid subsets we identified were indeed canine MDSC populations. However, legitimately labeling the cells as MDSCs would require evidence of their suppressive activity, prompting functional studies *in vitro*. For this purpose, we focused on the most numerous of the two myeloid populations, i.e. the CADO48A^+^ cells that were putatively PMN-MDSCs, to ensure that we would have sufficient cells available to allow reliable suppression assays to be performed.

### Hypodense CADO48A^+^ myeloid cells suppress CD8^+^ T cell proliferation *in vitro*

**Figure 2:**
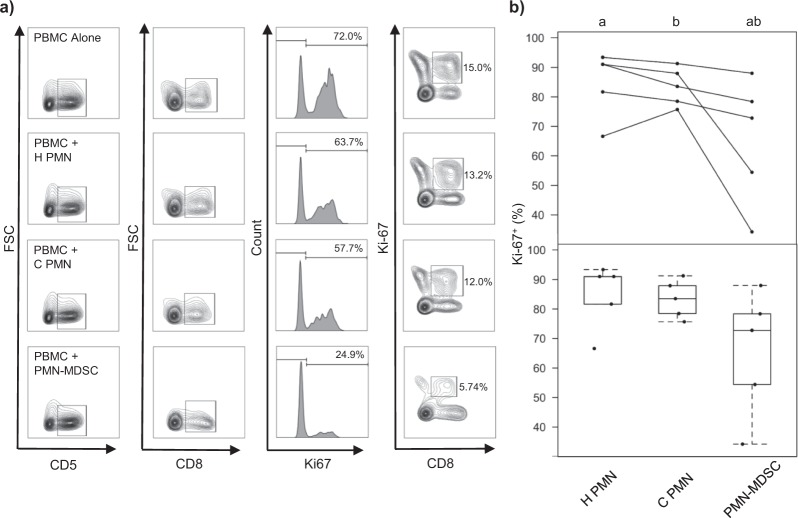
Canine CADO48A^+^ myeloid cells exhibit T cell suppression. (**a**) Exemplar of Ki-67 incorporation in CD5^+^CD8^+^ cells after PBMCs were co-cultured alone or 1:1 with H PMNs, C PMNs, and PMN-MDSCs for 72 hours. Exemplar depicts results using PBMCs from one healthy control dog. (**b**) Summary of proliferation assay results using cells isolated from five tumor-bearing dogs (normalized to PBMCs alone, average of co-culture results with two different healthy PBMCs). Top: Mean proportion of T cells that are Ki67+ [+ as superscript], with each line identifying C PMNs and PMN-MDSCs from the same tumor-bearing dog, as well the H PMNs used in that experiment. Bottom: Box-and-whisker plots depicting arithmetic mean values (dots) for all experiments. In each case, the box shows the respective 25th and 75th percentiles, the line indicates the median value, and the whiskers stretch from the lowest to the highest data points still within 1.5 times the interquartile range of the respective lower and upper quartiles. A letter indicates a significant difference for that comparison (p < 0.01).

The CADO48A^+^ cells suppressed polyclonal CD8^+^ T cell proliferation as indicated by reduced Ki-67 expression (Fig. 2a), confirming their functional credentials as regulatory cells. PMNs isolated from tumor-bearing dogs showed no statistically significant suppressive activity (Fig. 2b). Our findings therefore confirmed the suppressive function of the CADO48A^+^ cells. Given both the phenotypic resemblance of these cells to PMN-MDSCs and their regulatory credentials, we were able to confirm their identity as PMN-MDSCs. We hypothesized that the CD14^+^ myeloid cells would also be suppressive, and thus tentatively labeled them as M-MDSCs; however, in the absence of evidence of suppressive function, this designation remained speculative. Further interrogation of the molecular phenotype of the myeloid cells followed, drawing comparisons with both human and murine MDSCs.

### Canine MDSC subsets show distinctive transcriptomic signatures

**Figure 3:**
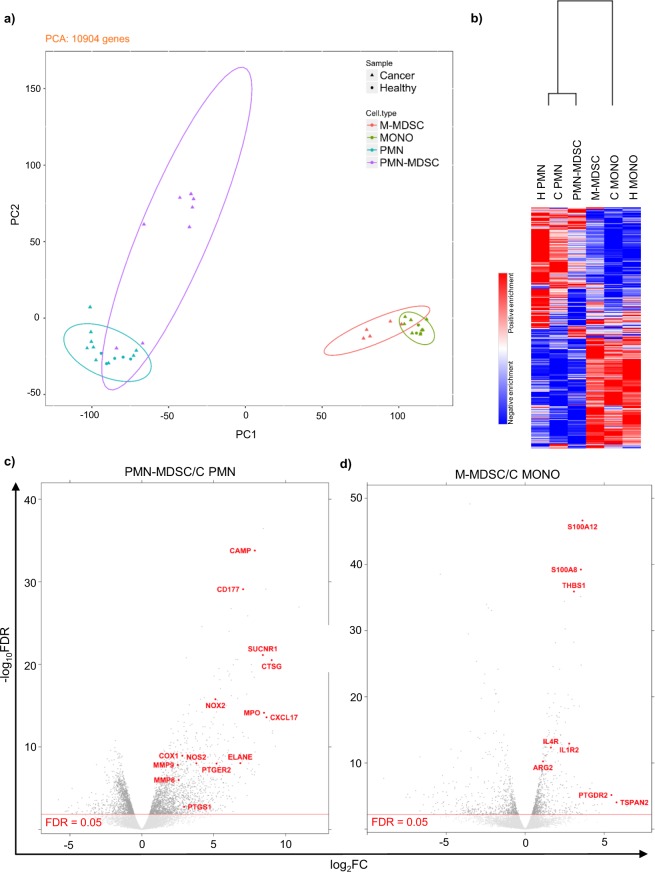
Canine MDSC subsets are characterized by distinct transcriptomic signatures. (**a**) Principal component analysis depicting transcriptional similarities of the different cell populations (demarcated by colors) and whether the sample came from a healthy (circle) or tumor-bearing (triangle) dog. Each symbol indicates one dog. The ellipse summarizes each cell subtype grouping using the multivariate t-distribution. PC1 accounted for 63.8% of all variance, while PC2 accounted for 8.22% of all variance. (**b**) Heatmap comparing transcriptional signatures across the 6 different cell populations, using the 1-Spearman rank correlation metric. Red indicates positive enrichment, and blue indicates negative enrichment. Increased vertical length of the dendrogram is inversely proportional to similarity between the cell types. (**c**,**d**) Volcano plot depicting differentially expressed genes in PMN-MDSCs relative to C PMNs (**c**) and putative M-MDSCs relative to C MONO MONOs (**d**). The red line indicates a significant FDR. Each dot represents one gene, and genes previously associated with MDSC function or novel but strongly upregulated genes are labeled in red.

We undertook RNA-Seq of PMN-MDSCs (n = 8), M-MDSCs (n = 5), PMNs (n = 9), and MONOs (n = 8) isolated from tumor-bearing dogs, and PMNs (n = 5) and MONOs (n = 3) isolated from healthy dogs. Principal component analysis of the samples from all dogs suggest the distinctive transcriptomes of the MDSC subsets, which were more closely related to their respective conventional populations than to each other (Fig. 3a). Similar conclusions were derived from a heatmap analysis comparing PMN-MDSCs, M-MDSCs, PMNs isolated from healthy (H PMN) and tumor-bearing patients (C PMN), and MONOs isolated from healthy (H MONO) and tumor-bearing patients (C MONO), supporting the finding that putative M-MDSCs, C MONOs, and H MONOs were more closely related to each other than to PMN-MDSCs, C PMNs, and H PMNs, which occupied a separate cluster (Fig. 3b). Within each cluster the respective MDSC subset was distinct, confirming its molecular credentials as a separate population from the adjacent conventional cells.

On deeper scrutiny of the different patterns of gene expression between cell populations, we observed that canine PMN-MDSCs differentially expressed a number of transcripts associated with MDSC function compared to C PMNs, including those encoding the matrix metalloproteases MMP8 and MMP9, nitric oxide synthase 2 (NOS2), NADPH oxidase 2 (NOX2), myeloperoxidase (MPO), cyclooxygenase 1 (COX1), the chemokine CXCL17, prostaglandin E synthase (PTGS1) and prostaglandin E receptor 2 (PTGER2) (Fig. 3c). A number of transcripts previously not attributed to PMN-MDSC function were also expressed at higher levels, including those encoding succinate receptor 1 (SUCNR1), cathelicidin antimicrobial peptide (CAMP), and cathepsin G (CTSG). Similar observations were made in putative canine M-MDSCs, which expressed transcripts encoding the calcium-binding proteins S100A8 and S100A12 at high levels compared to C MONOs, in addition to tetraspanin 2 (TSPAN2), IL1 receptor 2 (IL1R2), and IL4 receptor (IL4R) (Fig. 3d). Of interest, the respective PMN and MONO populations showed a number of differentially expressed genes when comparing tumor-bearing and healthy dogs (Supplementary Figs S2A–S2D).

**Figure 4:**
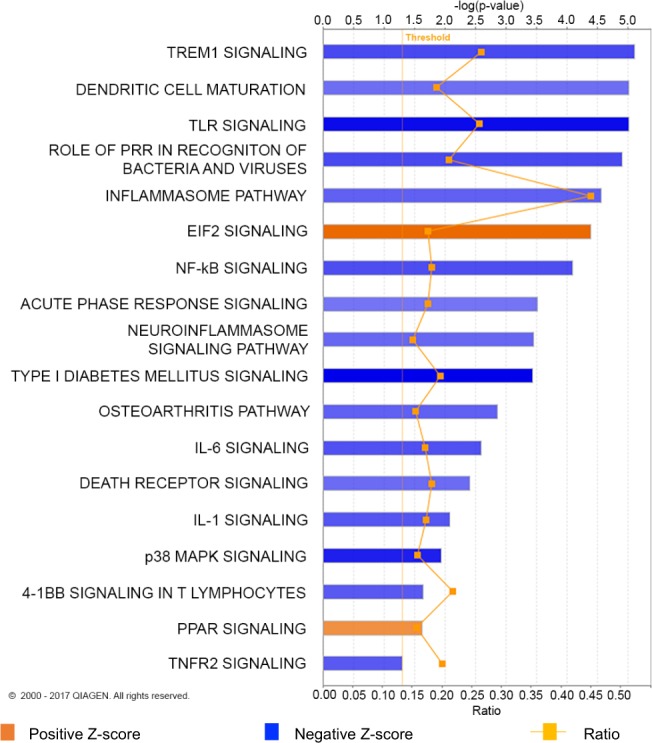
Canine PMN-MDSC transcriptomic signatures show enrichment of immune-related pathways. Ingenuity Pathway Analysis (IPA) of differentially expressed genes in PMN-MDSCs relative to C PMNs. Only those genes with a |FC| ≥ 2 and FDR ≤ 0.05 were imported into IPA. Greater color intensity correlates with stronger enrichment of Z-score, positive (orange) or negative (blue). Ratio refers to the proportion of genes represented in our data set relative to all known genes in IPA’s database for a given pathway.

Ingenuity Pathway Analysis was performed on all differentially expressed genes in PMN-MDSCs compared to C PMNs with an absolute FC ≥ 2 and FDR ≤ 0.05. Two pathways were found to be positively enriched in PMN-MDSCs, eukaryotic initiation factor 2 (EIF2) signaling and peroxisome proliferator-activated receptor (PPAR) signaling, while numerous pro-inflammatory pathways were negatively represented (Fig. 4).

Taken together, these findings substantiated our view that these hypodense myeloid populations were indeed MDSC subsets that were distinct from the comparative conventional cells.

### Cross-species analysis of PMN-MDSCs identifies a conserved gene signature

Focusing on the more numerous PMN-MDSC population, for which we had functional data, we next formally assessed the degree of similarity of the respective canine, human, and murine cells of this phenotype. We did not perform parallel analyses for our putative M-MDSC population since we have not yet validated their identity by T cell suppression assays.

**Figure 5:**
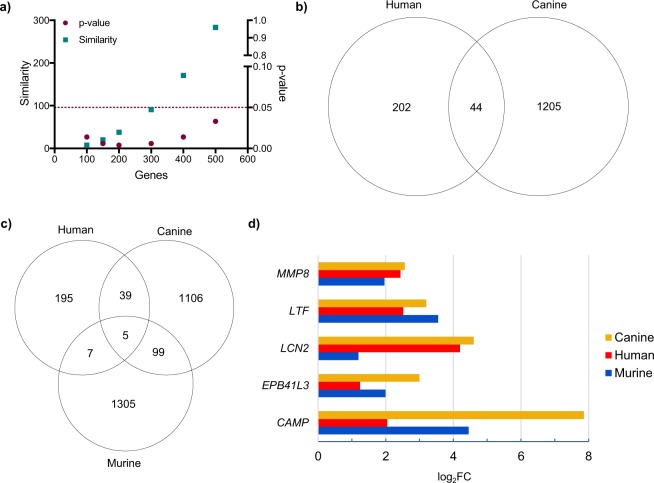
Cross-species comparisons of PMN-MDSCs highlight common transcriptomic signatures across three mammalian taxa. (**a**) Similarity score (square) and p-value (circle) are graphed using the top 100–500 canine and human PMN-MDSC differentially expressed genes input into the ‘OrderedList’ R program. Dashed line indicates p = 0.05, and up to the top 500 genes are statistically similar in both species. (**b**,**c**) Venn diagram comparing upregulated genes in canine and human (**b**) and canine, human, and murine (**c**) PMN-MDSCs relative to C PMNs. Only those genes with a FC ≥ 2 and FDR ≤ 0.05 were included in this analysis. (**d**) Bar chart comparing the fold change of the five conserved genes across the three species.

We compared our canine RNA-Seq data with those previously published on human and murine PMN-MDSCs. For all three species, genes differentially expressed by PMN-MDSCs compared to C PMNs were ordered according to the FDR of their expression, using only those genes that were detected in all three species. The resulting lists for each species were then analyzed for similarity. The signatures of the top 500 differentially expressed genes were statistically similar between canine and human PMN-MDSCs (Fig. 5a), but no such pattern was observed when this same list of genes were compared between canine and murine PMN-MDSCs (Supplementary Fig. S3A,B). To further understand the similarity between canine and human PMN-MDSCs, we identified those genes differentially expressed by PMN-MDSCs in comparison to C PMNs of both species with a FC ≥ 2. A comparison of these two lists identified 44 genes, including MPO, ELANE, as well as numerous ribosomal proteins (Fig. 5b and Supplementary Fig. S3C). Pathway analysis of these 44 genes highlighted six enriched pathways in PMN-MDSCs, including EIF2 signaling and TREM1 signaling (Supplementary Fig. S3D).

A comparison of those genes differentially expressed by PMN-MDSCs compared to C PMNs of dogs, humans and mice revealed five genes shared by all three species (Fig. 5c) – MMP8, LTF, LCN2, EPB41L3, and CAMP – four of which had not previously been implicated in PMN-MDSC function. A comparison of the FC expression in each species for these five genes, both in the respective transcriptomic datasets – including our own RNA-Seq dataset – and confirmatory RT-qPCR assays, revealed broad concordance (Fig. 5d and Supplementary Fig. S3E).

## Discussion

Pet dogs with spontaneous tumors have contributed to the development of a variety of therapies used in human medicine^11^. Their outbred nature, larger size, presence of an intact immune system, and exposure to environmental factors shared with humans makes them an attractive model to better understand cancer development, progression, and treatment^11,13^. Although previous studies in dogs with spontaneous tumors have identified MDSCs, these studies did not differentiate between the two established subsets of MDSCs^15,16^. Our study therefore set out to characterize the cellular and molecular phenotype of PMN-MDSCs and M-MDSCs in dogs, with the ultimate aim of better understanding these two cell populations and their role in cancer.

We identified myeloid populations in canine peripheral blood resembling both murine and human MDSCs, using a gating schema similar to that used to identify these cells in humans^4,6,33^. The lack of availability of canine-specific or validated cross-reactive mAb against CD15, CD66b and/or LOX-1 in dogs at the time of the current study precluded the use of these markers in our panel for PMN-MDSCs. Nevertheless, we were able to capitalize on the availability of a validated mAb used to identify PMNs in dogs^38^, which we substituted as a sentinel for CD15. This mAb was also used in one of the two original reports describing MDSCs in dogs^16^. The hypodense myeloid cells isolated using this mAb clearly resembled PMN-MDSCs by light and electron microscopy, thus lending support to their identity as PMN-MDSCs. In the case of M-MDSCs, the availability of a validated cross-reactive mAb against canine CD14^39^ facilitated the identification of this subset in a directly comparable manner to that of human M-MDSCs. Given that CD14 may also be expressed by non-myeloid cells^40^, we considered it important to adopt a cascaded gating approach focusing only on CD5^−^CD21^−^CD11b^+^ cells in our analyses. Electron microscopic images added additional weight to the putative identity of the MDSC subsets, demonstrating greater ER dilation than the respective conventional cells^17^. Further confirmation of the identify of these subsets came from our downstream transcriptomic studies.

Significantly higher frequencies of these MDSC subsets were documented in the peripheral blood of tumor-bearing dogs than healthy controls, showing parallels to findings in human patients^41,42^. Furthermore, bulkier tumors of a variety of histotypes were associated with higher frequencies of PMN-MDSCs – the more prevalent of the two populations in peripheral blood – concordant with the prevailing viewpoint that inflammatory signals generated in the tumor, which would broadly correlate with tumor size, drive the differentiation of MDSCs^43,44^. This phenomenon is also consistent with studies showing that tumor resection reduces^45,46^, and tumor recurrence increases^47^, MDSC frequency. Ultimate proof that the myeloid cells identified in our study were indeed PMN-MDSCs came with demonstration of their suppressive function, using a standard, polyclonal T cell suppression assay^48^. Variability of the suppression data reflected the clinical nature of samples, derived from individual dogs with various cancer histotypes. Moreover, PMN-MDSCs typically demonstrate only modest suppressive function that diminishes with age of sample in the context of polyclonal assays *in vitro*^4,6^, prompting our wish to streamline assays and minimize post-sorting delays before cells were put into culture. We therefore employed an assay whose readout was Ki-67 expression, which correlates with proliferation in multiple different contexts and shows greater sensitivity and ease of use than cell tracer dyes^49,50^. Furthermore, preliminary studies confirmed the close correlation between Ki-67 expression and dye dilution as metrics of T cell proliferation in our hands (data not shown).

Deeper analysis of the phenotype of the canine MDSC subsets by RNA-Seq yielded a number of interesting observations. Whether or not MDSCs are indeed a separate subset of cells has been a controversial topic in the field for a number of years^2,4^. Our transcriptomic findings suggested that they possess distinct molecular signatures, as shown by PCA and heatmaps of the respective populations. The canine MDSCs showed increased expression of a number of canonical genes implicated in MDSC function. For example, PMN-MDSCs produce reactive oxygen and nitrogen species^51,52^: in line with these characteristics, the canine PMN-MDSCs showed greater abundance of transcripts encoding NOS2, NOX2, and MPO than PMNs from the same patient^3,51,53^. COX1, which mediates the synthesis of prostaglandins and has been implicated in MDSC development^54,55^, was also differentially represented in canine PMN-MDSCs, as were PTGS1 and PTGER2. Furthermore, transcripts encoding the matrix metalloproteases MMP8 and MMP9 were differentially expressed by canine PMN-MDSCs. Functioning in degrading the extracellular matrix, these enzymes mediate the release of vascular endothelial growth factor (VEGF) to promote new vessel development and metastasis^56,57^. Interestingly, the transcript encoding CD177, a PMN marker^58,59^ expressed on the surface of MDSCs as well as MDSC exosomes^60^, was also differentially expressed by canine PMN-MDSCs. This observation prompted consideration of CD177 as a marker to distinguish canine PMN-MDSCs from PMNs. Increased expression of CXCL17 by PMN-MDSCs was also of interest, as previous studies have implicated CXCL17 in the recruitment of MDSCs and poor prognosis in human cancer patients^61,62^. Another differentially expressed transcript, SUCNR1, has been implicated in prostaglandin synthesis and promotion of an anti-inflammatory phenotype in a model of multiple sclerosis^63^; a similar mechanism may be at play in PMN-MDSCs. On the other hand, putative M-MDSCs showed greater abundance of transcripts encoding both the IL4R, consistent with previous data^8,64^, and S100A8, serum concentrations of which have been associated with poor cancer survival^36,65–67^. IL1R2 was also of interest, as it has previously been implicated in the transcription of IL-6 and VEGF, promoting angiogenesis^68^. Putative canine M-MDSCs differentially expressed transcripts encoding thrombospondin 1 (THBS1), another protein implicated in MDSC migration^60^, and TPSAN1, which has been implicated in cell invasion and motility in tumor cells^69^. Whether high expression of TPSAN1 by M-MDSCs could promote tumor progression was an intriguing question. IPA revealed additional interesting insights. One of the two sole pathways enriched in canine PMN-MDSCs – EIF2 signaling – has been previously reported to be enriched in human MDSCs^30^ and is induced by ER stress^30,70^, concordant with our ultrastructural analyses of these cells. PPAR signaling, meanwhile, has been implicated in the recruitment and expansion of MDSCs by a variety of extrinsic mechanisms^71^, but intrinsic roles for this signaling axis in MDSCs have hitherto not been recognized. Nevertheless, this pathway has anti-inflammatory properties in myeloid cells in general^72–74^, speaking to a possible role in maintaining the suppressive status of MDSCs. In contrast to EIF2 and PPAR signaling, a number of pro-inflammatory pathways were less active in canine PMN-MDSCs than C PMNs, consistent with the prevailing view of MDSCs as immunosuppressive cells. Taken together, the canine transcriptomic data therefore reconciled with previously published findings on these MDSC subsets in other species, while also revealing some novel avenues for future research.

Harnessing previously published transcriptomic data for humans and mice, we were surprised to discover that only the human and canine PMN-MDSCs had a significantly similar pattern of differentially expressed genes, adding support to the notion that the dog provides an attractive model for human cancer^11,13,75,76^. However, owing to the relative lack of available transcriptomic data in other species, these comparisons were made using only one human and one murine publication, both of which were based on gene microarray experiments. This difference in technical platform from our analysis had the potential to introduce bias into the comparisons, adding a note of caution to these preliminary findings. Nevertheless, using this published dataset to compare canine to human PMN-MDSCs identified 44 commonly upregulated genes. Importantly, pathway enrichment analysis using the 44 shared genes highlighted six pathways that are enriched in PMN-MDSCs. Interestingly, the two pathways with the most significant activation states in our canine PMN-MDSCs (EIF2 signaling and TREM1 signaling) were also highly enriched in the 44 genes shared between the two species, emphasizing the importance of these pathways to MDSC function^30,77^. HIF1α has been implicated in the differentiation of MDSCs to suppressive tumor-associated macrophages^78,79^, while Gαi signaling is required for the activation of STAT3^80,81^, an important transcription factor in PMN-MDSC development and function^82,83^. Representation of the colorectal cancer metastasis signaling pathway presumably reflects the importance of these cells to the dissemination of colorectal cancer^84^, but this notion remains speculative in the absence of more direct evidence. Enrichment of the phagosome maturation pathway was also interesting, because to the best of our knowledge it has not previously been associated with MDSC function. Phagocytosis is a well-established phenomenon in PMNs^85^, raising the important question of whether PMN-MDSCs also show phagocytic function, perhaps as an anti-microbial defense mechanism.

To further drill down on potential evolutionarily conserved mechanisms, we asked whether there were genes commonly upregulated in the PMN-MDSCs of all three species; five such genes were identified. Four of the five genes have not previously been implicated in PMN-MDSC function, and three of them – LTF, LCN2, and CAMP – play an important antimicrobial role^86,87^. LCN2 and LTF are both iron-binding proteins, raising the question of whether iron is important for MDSC suppressive function. Interestingly, LCN2 expands regulatory T cells^88^ and deactivates macrophages^89^, and LCN2 and LTF have been shown to play a protective role against oxidative stress^90,91^. Furthermore, LTF has been shown to inhibit neutrophil and eosinophil migration^92,93^. These novel observations, made by leveraging the power of evolution, therefore raise the intriguing possibility that PMN-MDSCs serve a hitherto unrecognized role in anti-microbial defenses, and/or have co-opted conserved antimicrobial peptides to serve immunoregulatory functions to promote tumor growth. In synopsis, both individual genes and evolutionarily conserved pathways shape the view that canine PMN-MDSCs show striking similarities to those of other mammalian species, but also raise important new questions about the functional breadth and versatility of these intriguing cells.

## Supplementary information

Supplementary Figures with Captions

Supplementary Figures with Captions, Changes Tracked

## Data Availability

Raw and processed data have been submitted to Gene Expression Ominbus (GEO).
