# Supplementary material for: Phenotypic and transcriptomic characterization of canine myeloid-derived suppressor cells
Source: Sci Rep. 2019 Mar 5;9:3574. doi: 10.1038/s41598-019-40285-3 (PMC6400936; doi:10.1038/s41598-019-40285-3)
Supplement: Supplementary file 1 — Supplementary Figures with Captions [file 41598_2019_40285_MOESM1_ESM.pdf]

SUPPLEMENTARY FIGURES

Phenotypic and transcriptomic characterization of canine myeloid-derived suppressor cells

Michelle R. Goulart[1,ξ,¶], Sabina I. Hlavaty[2,¶], Yu-Mei Chang[1], Gerry Polton[3], Anneliese Stell[1], James Perry[2], Ying Wu[1], Eshita Sharma[4], John Broxholme[4], Avery C. Lee[5], Balazs Szladovits[1], Mark Turmaine[6], John Gribben[7], Dong Xia[1,¥], Oliver A. Garden[2,¥,*]

[1]Royal Veterinary College, London, UK; [2]University of Pennsylvania School of Veterinary Medicine, Philadelphia, PA, USA; [3]North Downs Specialist Referrals, Surrey, UK; [4]Wellcome Centre for Human Genetics, University of Oxford, Oxford, UK; [5]University of Pennsylvania Perelman School of Medicine, Philadelphia, PA, USA; [6]Division of Bioscience, University College London, London, UK; [7]Barts Cancer Institute, London, UK

[¶]These authors contributed equally to this work

[ξ] Current address: Barts Cancer Institute, Queen Mary University of London, London, UK

[¥] Co-senior authorship

*Corresponding author: ogarden@upenn.edu

a)

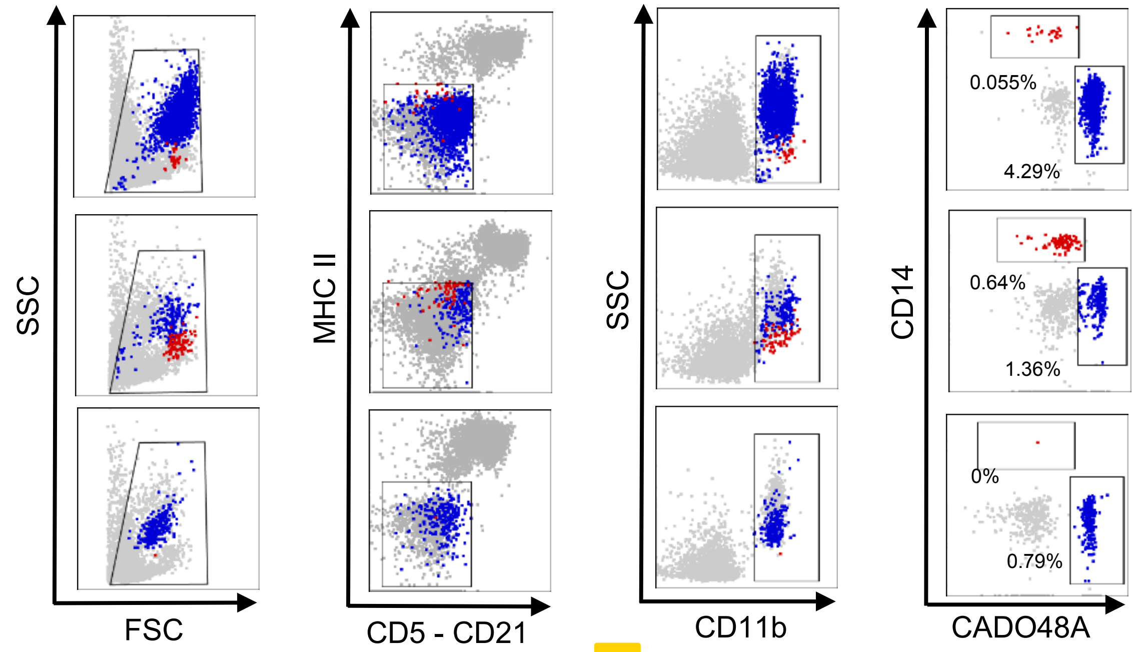
SSC
FSC
MHC II
CD5 - CD21
SSC
CD11b
CD14
CADO48A
0.055%
4.29%
0.64%
1.36%
0%
0.79%

b)

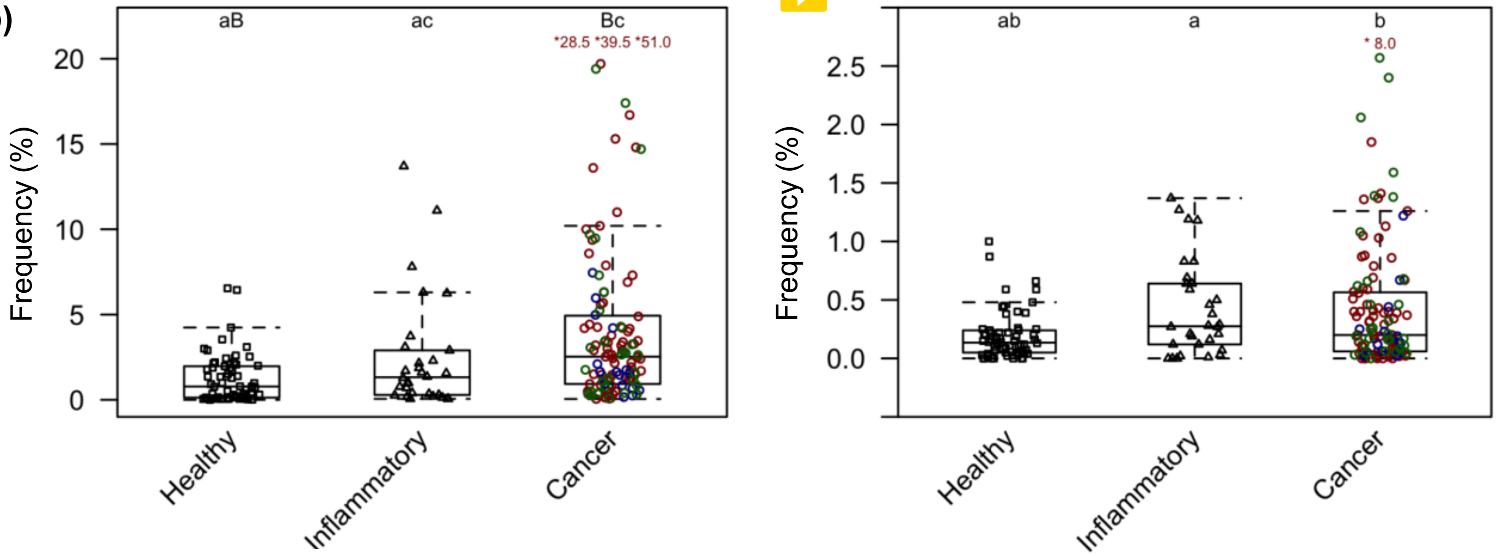
Frequency (%)
aB
ac
Bc
*28.5 *39.5 *51.0
Healthy
Inflammatory
Cancer
Frequency (%)
ab
a
b
* 8.0
Healthy
Inflammatory
Cancer

d)

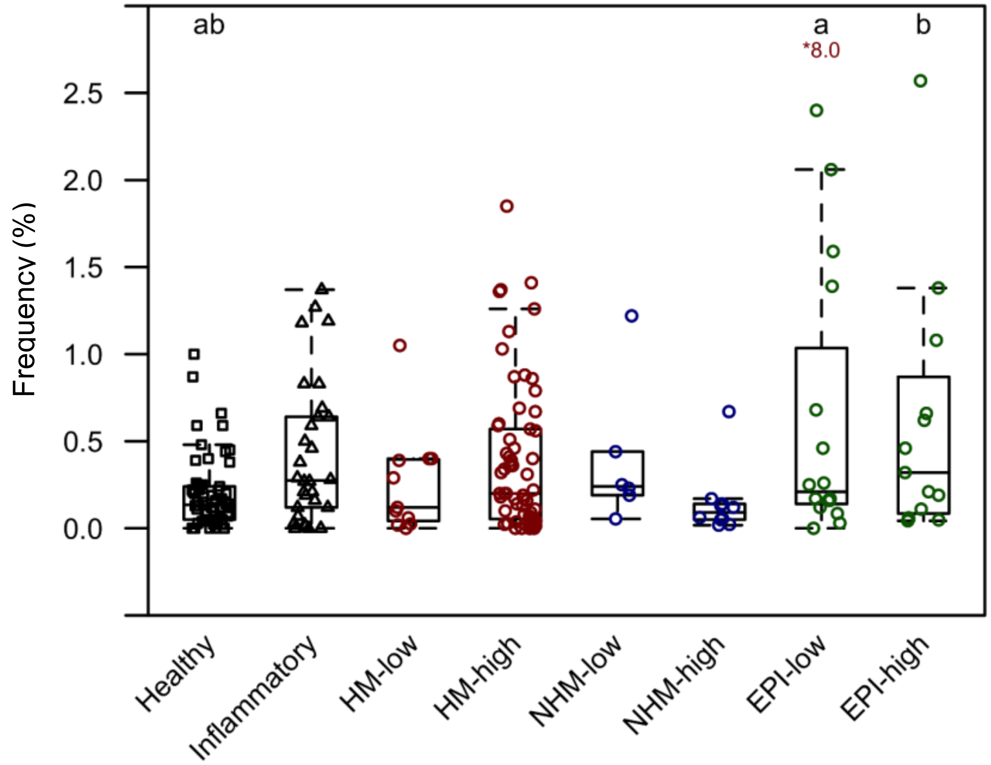
Frequency (%)
ab
a
*8.0
b
Healthy
Inflammatory
HM-low
HM-high
NHM-low
NHM-high
EPI-low
EPI-high

Supplementary Figure S1. Frequencies of putative MDSCs differ in tumor-bearing dogs compared to healthy and inflammatory control dogs

a) Parental gating of M-MDSCs (red dots) and PMN-MDSCs (blue dots) for the cascaded gating strategy used to identify both subsets in canine PBMCs. b) and c) Box-and-whisker plots depicting frequency of PMN-MDSCs (b) and M-MDSCs (c) in peripheral blood of canine healthy (square) and inflammatory (triangle) controls as well as tumor-bearing (circle) dogs. Frequencies are expressed as a percentage of total PBMCs. A capital letter denotes that the two groups are significantly different with $p < 0.001$, while a lowercase letter denotes a significant difference with $p < 0.05$. The red numbers represent dogs with frequencies off scale. The box represents quartiles, while the whisker-ends indicate the 5th and 95th percentile. Each dot represents a single dog, and the color indicates tumor histotype for tumor-bearing dogs (red: HM (hematopoietic mesenchymal), blue: NHM (non-hematopoietic mesenchymal), green: EPI (epithelial)). PMN-MDSCs: Healthy – 0.780% [0.133, 1.948], Inflammatory – 1.325% [0.305, 2.75]), Cancer – 2.53% [0.93, 4.935]), M-MDSCs: Healthy – 0.135% [0.05, 0.235], Inflammatory – 0.275% [0.12, 0.64], Cancer – 0.2% [0.06, 0.565]. d) Box-and-whisker plots depicting the frequency of PMN-MDSCs in peripheral blood of canine healthy and inflammatory controls as well as tumor-bearing dogs, grouped by cancer subtype (HM: hematopoietic mesenchymal, NHM: non-hematopoietic mesenchymal, EPI: epithelial) and burden (low vs high). Frequencies are expressed as a percentage of total PBMCs. A lowercase letter denotes a significant difference with $p < 0.01$. The box represents quartiles, while the whisker-ends indicate the 5th and 95th percentile. Each dot represents a single dog.

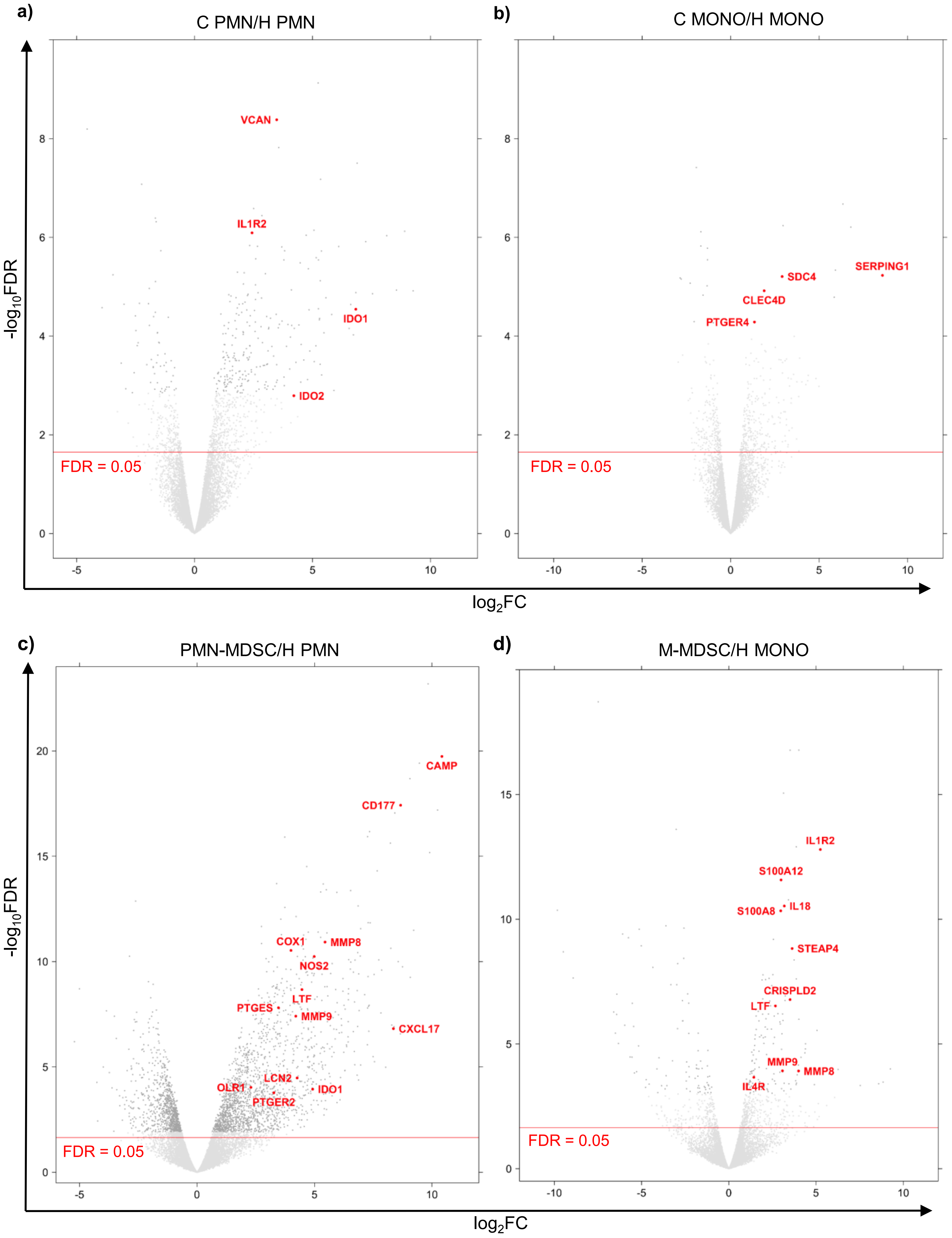
a)
C PMN/H PMN
VCAN
IL1R2
IDO1
IDO2
FDR = 0.05
b)
C MONO/H MONO
SERPING1
SDC4
CLEC4D
PTGER4
FDR = 0.05
-log10FDR
log2FC
c)
PMN-MDSC/H PMN
CAMP
CD177
COX1
MMP8
NOS2
LTF
PTGES
MMP9
CXCL17
LCN2
OLR1
IDO1
PTGER2
FDR = 0.05
d)
M-MDSC/H MONO
IL1R2
S100A12
S100A8
IL18
STEAP4
CRISPLD2
LTF
MMP9
MMP8
IL4R
FDR = 0.05
-log10FDR
log2FC

Supplementary Figure S2. Canine MDSC subsets and PMNs are characterized by distinct transcriptomic signatures

a-d) Volcano plots depicting differentially expressed genes in the indicated cell populations: C PMNs compared to H PMNs (a), C MONOs compared to H MONOs (b), PMN-MDSCs compared to H PMNs (c), and putative M-MDSCs compared to H MONOs (d). The red line indicates a significant FDR. Each dot represents one gene, and genes previously associated with MDSC function or novel but strongly upregulated and downregulated genes are labeled in red.

a)

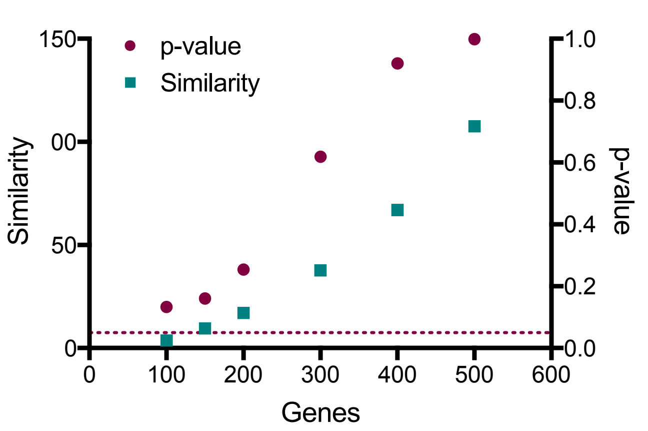

b)

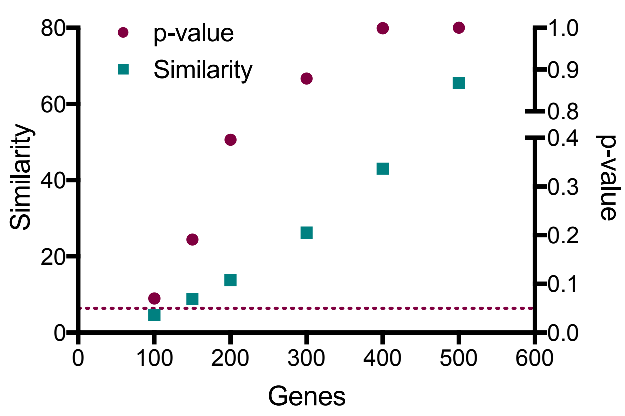

c)

Shared Upregulated Genes In Human and Canine PMN-MDSCs (relative to C PMN)			
CTSG	*TCN1*	*S100A10*	*CD86*
MPO	*AKR1A1*	*STOM*	*RPS17*
CAMP	*RGS10*	*GPI*	*RPS4X*
ELANE	*ABCA13*	*PRDX1*	*RPS5*
GNG11	*CYFIP1*	*CD74*	*RCC2*
PTGER2	*LTF*	*XPNPEP1*	*RPSA*
PPBP	*EPB41L3*	*LGALS1*	*RPL15*
BPI	*MMD*	*RPL3*	*RPS12*
SLC2A5	*METAP2*	*AHNAK*	*RPL5*
GP9	*MMP8*	*PPA1*	*RPL35*
LCN2	*FGD2*	*F13A1*	*PRNP*

d)

Ingenuity Canonical Pathways	-log(p-value)	Ratio ($x10^{-2}$)	Molecules
EIF2 Signaling	9.31	4.07	RPL15, RPL35, RPL3, RPL5, RPS17, RPS5, RPS12, RPS4X, RPSA
Phagosome Maturation	2.48	2.03	MPO, PRDX1, CTSG
TREM1 Signaling	2.00	2.67	MPO, CD86
Colorectal Cancer Metastasis Signaling	1.87	1.21	GNG11, MMP8, PTGER2
HIF1α Signaling	1.63	1.69	SLC2A5, MMP8
Gαi Signaling	1.61	1.67	GNG11, RGS10

e)

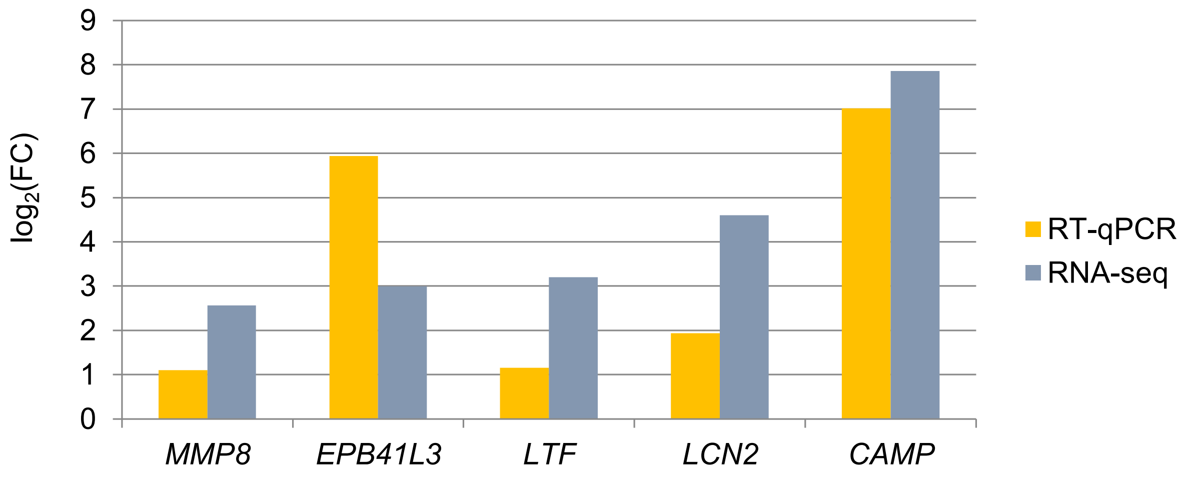

Supplementary Figure S3. Cross-species analysis highlights genes differentially expressed in PMN-MDSCs shared by multiple mammalian taxa

a) and b) Similarity score (square) and p-value (circle) are graphed using the top 100–500 differentially expressed genes in human and murine (a) and canine and murine (b) PMN-MDSCs relative to C PMNs. Dashed line indicates $p = 0.05$. c) Table listing the 44 shared upregulated genes in human and canine PMN-MDSCs shown in Figure 3b, listed in order of decreasing FC in the canine data, starting at CTSG down from left to right. d) Pathway enrichment analysis performed in IPA on the gene list in (c). Ratio indicates the proportion of genes found in our dataset relative to total number of genes in that pathway in IPA. e) Validation of RNA-Seq results of the five interesting genes upregulated in canine PMN-MDSCs compared to canine C PMNs using RT-qPCR. FC data are based on the mean result of four biological replicates.
